# A thermostable GH45 endoglucanase from yeast: impact of its atypical multimodularity on activity

**DOI:** 10.1186/1475-2859-10-103

**Published:** 2011-12-06

**Authors:** Marie Couturier, Julia Feliu, Mireille Haon, David Navarro, Laurence Lesage-Meessen, Pedro M Coutinho, Jean-Guy Berrin

**Affiliations:** 1 INRA, UMR 1163 BCF, 13288 Marseille, France; 2 CNRS, AFMB, 13288 Marseille, France; 3 Aix-Marseille Universités, 13288 Marseille, France

**Keywords:** biomass, crystalline cellulose, cellulase, *Pichia pastoris*, thermostable GH45 endoglucanase

## Abstract

**Background:**

The gene encoding an atypical multi-modular glycoside hydrolase family 45 endoglucanase bearing five different family 1 carbohydrate binding modules (CBM1), designated *Pp*Cel45A, was identified in the *Pichia pastoris *GS115 genome.

**Results:**

*Pp*Cel45A (full-length open reading frame), and three derived constructs comprising (i) the catalytic module with its proximal CBM1, (ii) the catalytic module only, and (iii) the five CBM1 modules without catalytic module, were successfully expressed to high yields (up to 2 grams per litre of culture) in *P. pastoris *X33. Although the constructs containing the catalytic module displayed similar activities towards a range of glucans, comparison of their biochemical characteristics revealed striking differences. We observed a high thermostability of *Pp*Cel45A (Half life time of 6 h at 80°C), which decreased with the removal of CBMs and glycosylated linkers. However, both binding to crystalline cellulose and hydrolysis of crystalline cellulose and cellohexaose were substantially boosted by the presence of one CBM rather than five.

**Conclusions:**

The present study has revealed the specific features of the first characterized endo β-1,4 glucanase from yeast, whose thermostability is promising for biotechnological applications related to the saccharification of lignocellulosic biomass such as consolidated bioprocessing.

## Background

Lignocellulosic biomass is the largest renewable source of carbohydrates for the production of biofuels, biomaterials, and high-value products but its recalcitrance to enzymatic degradation makes industrial processes complex and costly [1,2]. The main component of plant cell wall is cellulose, a linear polymer of β-1,4 linked glucose units. Cellulose chains are arranged in linear microfibrils that form very recalcitrant crystalline-like structures, and form a tight complex with varying proportions of hemicellulose and lignin [3]. The hydrolysis of cellulose into glucose monomers requires the coordinated action of several types of complementary enzymes: endoglucanases (endo-β-1,4-glucanases, EC 3.2.1.4) randomly cleaving glycosidic bonds on cellulose polymers, cellobiohydrolases (cellulose β-1,4-cellobiosidases, EC 3.2.1.94) sequentially releasing cellobiose from cellulose chain ends, and β-glucosidases (β-1,4-glucosidases, EC 3.2.1.21) converting cellobiose into glucose monomers [4]. More recently, other types of enzymes, e.g. GH61, have been reported as able to enhance the conversion of cellulose when used in conjunction with a cellulase or a mixture of cellulases [5]. They may contribute to cellulose degradation using a different mode of action [6].

Among the 128 glycoside hydrolases (GHs) families (CAZy - [7,8]), fungal endoglucanases are grouped, along with other enzymes, into 8 GH families, including GH5, GH6, GH7, GH9, GH12, GH45, GH48, and GH74. Family GH45 endoglucanases are distantly related to plant expansins and found in a broad range of organisms including bacteria, plants, animals, and fungi. They are characterized by a low molecular weight and an inverting stereochemical mechanism [9], and usually produce cello-oligosaccharides as end-products from cellulose substrates without any glucose release as reported for endoglucanases belonging to other families [10].

Many glycoside hydrolases modular enzymes, where catalytic and non catalytic modules may be separated by linkers, are often highly glycosylated in fungi [11]. Carbohydrate binding modules (CBM), able to bind one or several type of polysaccharides, are found among the non-catalytic modules. The sugar-binding activity of CBMs increases the enzyme concentration in the vicinity of the substrates, leading to a more effective hydrolysis [12]. It has also been suggested that CBMs could play a role in the degradation of polysaccharides *via *a destructurative action on the substrate fibrils [13,14]. Amongst the 64 CBMs families classified in CAZy, CBM1s are almost exclusively found in fungi and specifically bind crystalline cellulose. They are characterized by the prevalence of aromatic amino acid residues in the binding surface that forms a platform-like architecture [15]. This planar organization of the binding site is thought to be complementary to the flat surfaces presented by cellulose crystals. CBMs can be localized at the N- or C-terminal end of the catalytic module alone or in multiple organizations [16]. The presence of multiple CBMs in a glycoside hydrolase is usually found in bacteria, such as in the α-amylase from *Lactobacillus amylovorus *that contains five CBM26 arranged in tandem [17]. To our knowledge, the only example of a characterized eukaryotic GH carrying multiple CBM1s modules is a GH45 endoglucanase from *Mucor circinelloides *which bears two CBM1 [18].

Upon analysis of the recently sequenced *Pichia pastoris *GS115 genome [19], we have identified an intriguing modular family 45 endoglucanase containing five different CBM1 modules arranged in tandem at the N-terminus. To investigate the role played by these multiple CBMs in the hydrolysis of cellulose, we homologously expressed the full length endoglucanase and three truncated derivatives, and further characterized them biochemically to evaluate their potential as tools for cellulose conversion.

## Results

### Bioinformatic analysis of *Pp*Cel45A

**Figure 1:**
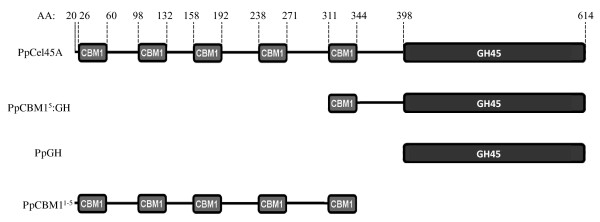
**Modular representation of *Pp*Cel45A and derived constructs used in the study**.

**Figure 2:**
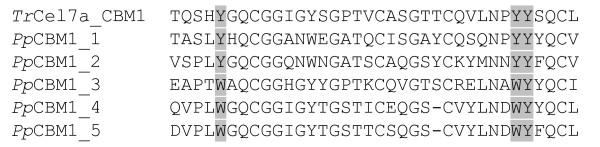
**Alignment of the 5 CBMs amino-acid sequences**. The three conserved aromatic amino acids (by referring to *T. reesei *Cel7a CBM1 sequence) forming a hydrophobic platform for interaction with cellulose are shown in grey.

**Figure 3:**
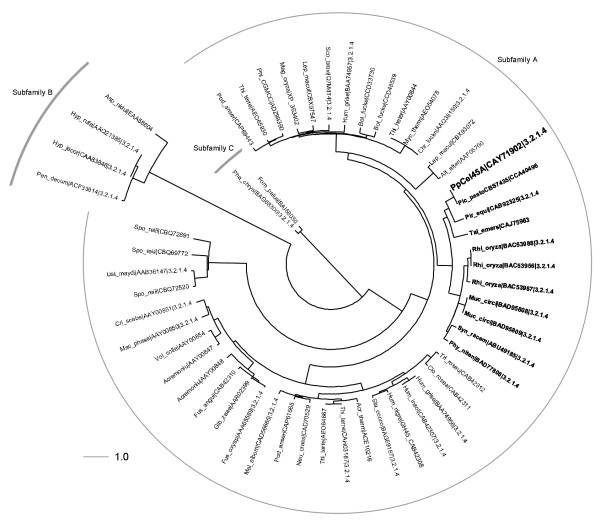
**Phylogenetic representation of family GH45 from fungal origin**. The cladogram highlights the relative position of the proteins labelled with the abbreviation of the species name, the reference public database accession numbers (Genbank) and the identified activity for characterized enzymes (EC numbers). Subfamilies are indicated based on [28].

*PpCel45A *is a 1,845 bp gene (defined as [Genbank:PAS_chr4_0643]) containing no identified introns that encodes five N-terminal CBM1 modules and one C-terminal family 45 endoglucanase module (Figure 1). The six modules are separated by linkers carrying numerous potential O-glycosylation sites (Ser and Thr). Comparison of the five amino-acid CBM1 sequences revealed that they were all different with a maximum identity of 91% between CBM1-1 and CBM1-2 and a minimum identity of 41% between CBM1-2 and CBM1-3 (Figure 2). Interestingly, the result of BLAST searches showed that *Pp*Cel45A is the first endo β-1,4 glucanase identified in ascomycete yeast. The X33 strain is derived from the GS115 strain and we suggest that it probably also contains PpCel45A, as does the *P. pastoris *CBS 7435 strain that was also sequenced recently. However, to our knowledge other *Pichia *strains (e.g. *P. stipitis *CBS 6054) do not have any family 45 endoglucanase. The *P. pastoris *GS115 GH45 module sequence was distantly related to *T. reesei *family 45 endoglucanase V/Cel45a (17% amino-acid identity) but had high identity with endo-ß-1,4-glucanases from the Mucorales order. Indeed, a phylogenetic analysis of 55 fungal GH45 was performed and revealed that *Pp*Cel45A and Mucorales GH45 are grouped together with *P. equi *Cel45A in GH45 subfamily A (Figure 3), whereas *T. reesei *Cel45a belongs to subfamily B. The high similarity between *Pp*Cel45A, *P. equi *Cel45A and Mucorales GH45 suggests that *Pp*Cel45A as well as *P. equi *Cel45A may have been acquired by horizontal gene transfer events. Horizontal gene transfer events have been described as common among the anaerobic fungi of the rumen [20], but to our knowledge such events have not been described between fungi and yeasts (Saccharomycetales).

### Cloning, expression, and purification of *Pp*Cel45A and derived constructs in *P. pastoris*

The DNA sequences encoding either the entire *P. pastoris Pp*Cel45A (full-length open reading frame) and three truncated derivatives, *Pp*CBM1^5^:GH (catalytic module with its proximal CBM1), *Pp*GH (catalytic module), and *Pp*CBM1^1-5 ^(five CBM1 modules without catalytic module) (Figure 1) were amplified by PCR using genomic DNA from *P. pastoris *GS115. Each sequence was inserted into the *P. pastoris *expression vector pPICZαA, in frame with the yeast α-factor secretion peptide and a (His)_6 _tag located at the C-terminus, and under the control of the *AOX1 *promoter as described in Materials and Methods. Full-length protein and truncated proteins were all successfully expressed in *P. pastoris *X33, i.e. they were visualized in the supernatant after 3-days induction using SDS-PAGE, indicating correct processing of the α-factor signal sequence. SDS-PAGE analysis showed that expression yield reached up to 2 g per litre of culture (data not shown). Only traces of endogenous proteins were detected in the culture supernatants of transformants (data not shown). Each selected transformant was scaled-up to increase the production of each recombinant protein. Purification was performed by affinity chromatography, taking advantage of C-term (His)_6 _tags. High production and purification yields were obtained with up to 250 mg of purified protein per litre of induced culture after three days induction (Table 1).

**Table 1:** Biochemical characterization of *Pp*Cel45A derived constructs

	Purification yield (mg/l) ^a^	Molecular mass (kDa) Theoretical Experimental ^b^		Apparent optimum pH	Apparent optimum temperature (°C) ^c^
*Pp*Cel45A	172	61.9	150	4.8	37-75 (65)
*Pp*CBM1^5^:GH	250	31.5	80	4.8	37-75 (65)
*Pp*GH	215	22.6	35	4.8	37-75 (65)
*Pp*CBM1^1-5^	68	35.2	134	n.d.	n.d.

^a ^expression yield is expressed in mg of recombinant protein purified per litre of culture

^b ^values are estimated from SDS-PAGE

^c ^values indicate that relative enzyme activity remaining was above 50%. Values in brackets indicate apparent optimum temperature under the conditions used

n.d. : not determined since *Pp*CBM1^1-5 ^displays no catalytic activity.

### Biochemical characterization

**Figure 4:**
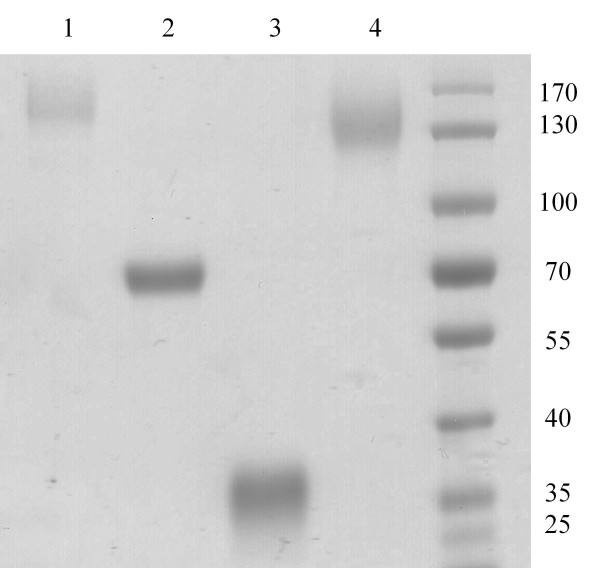
**SDS-PAGE analysis of *Pp*Cel45A and its derived constructs**. 10 μg of each purified recombinant protein was loaded onto a 12% Tris glycine SDS-PAGE and proteins were stained with Coomassie blue. *Pp*Cel45A (lane 1), *Pp*CBM1^5^:GH (lane 2), *Pp*GH (lane 3) and *Pp*CBM1^1-5 ^(lane 4). Molecular masses (kDa) of marker proteins are shown on the right.

**Figure 5:**
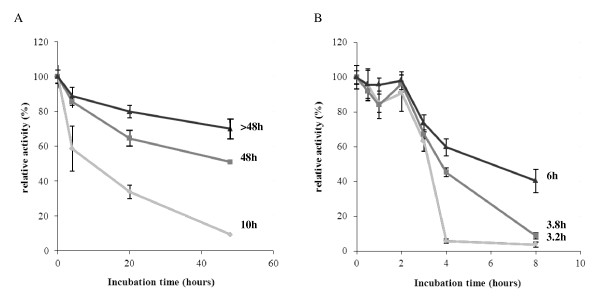
**Thermostability of *Pp*Cel45A and its derived constructs**. Thermostability profiles at 65°C (A) and 80°C (B). Light gray: *Pp*GH, dark gray: *Pp*CBM1^5^:GH, black: *Pp*Cel45A. Half-lives of the three constructions are indicated on the right.

The four purified recombinant proteins were analyzed on SDS-PAGE for assessment of apparent molecular weight (Figure 4, Table 1). They displayed apparent molecular weights higher than the theoretical ones, probably resulting from post-translational modifications [21]. *Pp*CBM1^1-5 ^displayed no enzymatic activity under all the conditions tested. Apparent optimum pH and temperature were determined on *Pp*Cel45A, *Pp*GH, and *Pp*CBM1^5^:GH. All three exhibited the same pH and temperature profile with an optimum pH at 4.8 and more than 50% activity between pH 3 and 8. Their optimum temperature was 65°C, with more than 50% activity between 30°C and 70°C (Table 1). Thermal stability was also evaluated on the three constructions at 65°C and 80°C. (Table 1 Figure 5). It revealed that *Pp*Cel45A was stable up to 80°C with a residual activity of 70% and 60% after 48 hours at 65°C and 4 hours at 80°C, respectively. *Pp*GH was less stable with 9% activity retained after 48 hours at 65°C and 6% after 4 hours at 80°C. *Pp*CBM1^5^:GH was more stable than *Pp*GH with respectively 51% and 45% residual activity after 48 hours at 65°C and 4 hours at 80°C, respectively.

### Catalytic properties of *Pp*Cel45A

**Figure 6:**
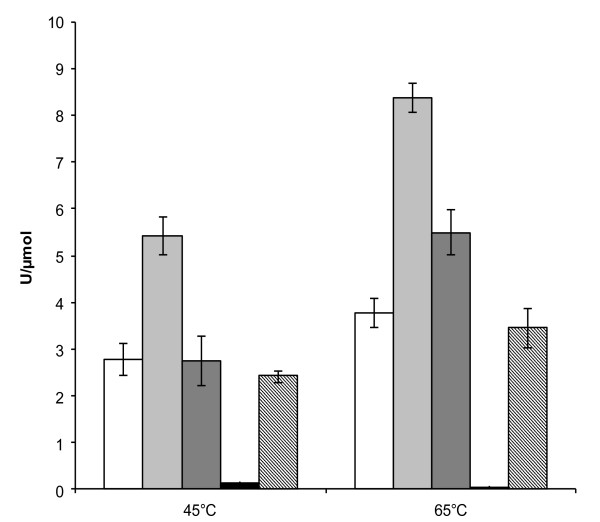
**Hydrolysis of crystalline cellulose at different temperatures**. Hydrolysis was performed on Avicel for 16 hours at 45°C or 65°C with: *Pp*GH (white bars), *Pp*CBM1^5^:GH (light gray bars), *Pp*Cel45A (dark gray bars), *Pp*CBM1^1-5 ^(black bars) and *Pp*GH+*Pp*CBM1^1-5 ^in equimolar amounts (striped bars).

The four constructions were evaluated for their ability to degrade a wide range of cellulosic and hemicellulosic substrates (Table 2). Although *Pp*CBM1^1-5 ^had no detectable activity towards the substrates tested, the three other constructions (*Pp*GH, *Pp*CBM1^5^:GH, *Pp*Cel45A) displayed similar activities towards soluble cellulose derivatives (CMC and HEC), β-1,3/β-1,4 glucans (β-glucan and lichenan), and one hemicellulolytic substrate (konjac glucomannan), the highest activity being obtained using lichenan. However, no activity was detected using pNP-Cel, pNP-Lac, pNP-Glu, filter paper, mannan, and chitin, even after extensive hydrolysis. Avicel hydrolysis was performed for 16 hours at 45°C or 65°C (Figure 6). *Pp*CBM1^1-5 ^displayed no activity towards Avicel. *Pp*GH, *Pp*CBM1^5^:GH, and *Pp*Cel45A hydrolyzed Avicel significantly and more efficiently at 65°C, yielding specific activities of 3.8, 8.4 and 5.5 U/μmol. There was no improvement of Avicel hydrolysis using equimolar amount of *Pp*GH and *Pp*CBM1^1-5 ^compared to *Pp*GH alone. Although no difference was observed between *Pp*Cel45A and *Pp*GH at 45°C, *Pp*Cel45A was 1.5-fold more active than *Pp*GH at 65°C. Surprisingly, *Pp*CBM1^5^:GH was significantly more active than *Pp*GH and *Pp*Cel45A on Avicel both at 45°C and 65°C.

**Table 2:** Specific activities (U/μmol) of *Pp*Cel45A derived constructs on pNP substrates and polysaccharides from different sources

	Lichenan	β-glucan	CMC	Glucomannan	HEC	Avicel
*Pp*Cel45A	833 ± 34	203 ± 17	196 ± 16	190 ± 23	54 ± 2	2.74 ± 0.53
*Pp*CBM1^5^:GH	792 ± 27	123 ± 9	177 ± 13	188 ± 19	53 ± 5	5.43 ± 0.41
*Pp*GH	810 ± 29	124 ± 21	160 ± 2	161 ± 9	43 ± 4	2.77 ± 0.35
*Pp*CBM1^1-5^	ND	ND	ND	ND	ND	ND

Specific activities were determined at a substrate concentration of 10 mg.ml^-1 ^at 45°C in 50 mM citrate phosphate buffer pH 4.8 and expressed as units per μmol of enzyme in order to ensure comparison independently of molecular mass. Values are means of triplicate independent measures and standard errors of the mean are shown next to each value. N.D.: no activity detected.

### Analysis of end products

To evaluate the mode of action of *P. pastoris *endoglucanase, the soluble sugars generated upon hydrolysis of either crystalline cellulose, soluble cellulose or cello-oligosaccharides by the three active recombinant constructions were determined by anion exchange chromatography. The products formed upon hydrolysis of polymeric substrates (Avicel and CMC) were analyzed, showing similar product patterns for the three enzymes, i.e. the oligo-saccharides G4, G3, and G2 were produced in the first minutes of the reaction. Toward the end of the reaction, cellobiose (G2) and cellotriose (G3) were the main products detected (data not shown). Upon hydrolysis of soluble cellulose (CMC), the three enzymes yielded similar amounts of oligo-saccharides at the end of the reaction. However, when crystalline cellulose (as in Avicel) was used as a substrate, *Pp*Cel45A released two times more end-products than *Pp*GH, and CBM1^5^:GH almost three times more than *Pp*GH. The products formed upon hydrolysis of cellohexaose (G6), cellopentaose (G5), and cellotetraose (G4) were also analyzed. The three enzymes showed similar hydrolytic patterns towards cello-oligosaccharides. G6 was hydrolyzed to produce G4 and G2 with traces amounts of G3, and G5 was degraded to form exclusively G3 and G2. G4 was hydrolyzed to produce exclusively G2 but with a lower rate that circumvented the determination of the catalytic efficiency. Neither *Pp*Cel45A nor their truncated derivatives showed detectable hydrolysis on cellotriose. Initial rate data from hydrolyses of G6 and G5 at 40°C and pH 4.8 indicated that the catalytic efficiency (k_cat_/K_m_) increased with increasing chain length (Table 3). It is interesting to note that *Pp*CBM1^5^:GH catalytic efficiency towards G6 was significantly higher compared to *Pp*GH and *Pp*Cel45A.

**Table 3:** Specificity constants determination of *Pp*Cel45A, *Pp*CBM1^5^:GH and *Pp*GH on cello-oligosaccharides.

	*Pp*Cel45A	*Pp*CBM1^5^:GH	*Pp*GH
G6	1.35 × 10^4^	7.60 × 10^4^	3.8 × 10^4^
G5	6.3 × 10^3^	6.3 × 10^3^	8.1 × 10^3^
G4	n.d.	n.d.	n.d.

Specificity constants (k_cat_/K_m_) are expressed as min^-1^.M^-1^.

n.d. : not determined. Although enzyme activity was present, it was too low to measure the individual kinetic constant.

### Substrate binding capacities of *Pp*Cel45A and derived constructs

The ability of *Pp*Cel45A and its respective constructs to bind crystalline cellulose was investigated. Binding to Avicel was observed for *Pp*Cel45A, *Pp*CBM1^1-5^, *Pp*CBM1^5^:GH, but not for *Pp*GH. Based on typical double-reciprocal plots, binding parameters were determined for the three constructions, yielding *K_d _*values of 0.45, 0.78, and 0.95 for *Pp*CBM1^1-5^, *Pp*Cel45A, *Pp*CBM1^5^:GH, respectively (Table 4). The B_max _value of *Pp*CBM1^5^:GH (0.80 μmol per g of substrate) was 4 times higher compared to *Pp*CBM1^1-5 ^and *Pp*Cel45A (0.19 and 0.20 μmol per g of substrate, respectively).

**Table 4:** inding parameters of *Pp*Cel45A and derived constructs on crystalline cellulose

Enzyme components	*K_d _*(μM)	B_max _(μmol bound per g of substrate)
*Pp*Cel45A	0.45	0.199
*Pp*CBM1^5^:GH	0.95	0.794
*Pp*GH	n.d.	n.d.
*Pp*CBM1^1-5^	0.78	0.185

Binding parameters were determined using Avicel in duplicate and the *K_d _*and B_max _values were calculated using double-reciprocal plots. n.d. no binding detected.

## Discussion

Several family 45 endoglucanases have been characterized in the last years from fungi [9,22], bacteria [23], or animals [24]. However, to our knowledge the present study is the first report on the characterization of a GH45 from yeast. Since *P. pastoris *GS115 growth on cellulose as a sole carbon source was very limited, the function and origin of such an endoglucanase in yeast remains enigmatic and could be the result of a horizontal transfer from Mucorales. Since *P. pastoris *X33 has been proven to be an ideal host for the production of eukaryotic glycosidases [25-27], we successfully used it for homologous expression of *Pp*Cel45A and three truncated derivatives. The four proteins were successfully produced with high yields and purified in one step thus allowing their in depth characterization.

The highest specific activity displayed by the three active derivatives of *Pp*Cel45A was obtained toward lichenan, which has also been described as the best substrate for other GH45, as *Pc*Cel45A from *Phanerochaete chrysosporium *[28] or *Bx-*ENG-1, -2 and -3 from the pine wood nematode *Bursaphelenchus xylophilus *[29]. Although chitin and cellulose structures are similar, no activity of *Pp*Cel45A was detected toward chitin. Hydrolysis experiments using mannan and glucomannan revealed that *Pp*Cel45A was not capable to hydrolyze the β-1,4 linkages between mannosyl residues. However it hydrolyzed efficiently glucomannan suggesting that part of β-1,4 linkages found between mannose and glucose residues were efficiently hydrolysed. *Pp*Cel45A revealed typical classical endoglucanase features, i.e. efficient hydrolysis of soluble celluloses derivatives (CMC and HEC), slow hydrolysis of crystalline cellulose forms (Avicel) and no hydrolysis of pNP-Cel, rather than processive features. Although it has been suggested that carbohydrate binding modules could play a role in the destructuration of cellulose [14], *Pp*CBM1^1-5 ^displayed no identified activity toward any of the polysaccharides tested. Furthermore, the number of CBMs of *P. pastoris *endoglucanase did not influence the activity of *Pp*Cel45A on soluble substrates, in good agreement with Baba *et al*. [18].

The patterns of CMC, Avicel, and cello-oligosaccharides hydrolysis end products were similar for the three constructions. The major CMC end products were cellobiose and cellotriose, with trace amounts of glucose produced at the end of the reaction, as reported for the endoglucanase from *Penicillium decumbens *[22] but different from *T. reesei *Cel45A (EG V) that produced only trace of cellobiose from CMC [10]. The major Avicel end products were also cellobiose, cellotriose, and trace amounts of glucose, but the amounts of end products released varied depending on the enzyme component. *Pp*CBM1^5^:GH was therefore more efficient than *Pp*Cel45A, which was more efficient than *Pp*GH. No glucose was released from any of the oligosaccharides tested, confirming the endo mode of action of *Pp*Cel45A. End products profiles from hydrolyzed cello-oligosaccharides were in good agreement with previous studies [30,31] and suggest an organization of the catalytic site in six subsites. This hypothesis is strengthened by the crystal structure of *Humicola insolens *GH45 [9] that revealed six subsites in the active site, with an organization from -4 to +2 [32].

Thermostable hemicellulases and cellulases are gaining interest because they are well suited for harsh industrial process conditions [33]. Several thermostable endoglucanases have already been isolated from thermotolerant or thermophilic organisms like the GH45 endoglucanase from *Syncephalastrum racemosum *[34] and the Cel5A endoglucanase from *Thermoanaerobacter tengcongensis *[35]. Compared to other thermostable endoglucanases, *Pp*Cel45A thermal properties were remarkable since *P. pastoris *is a mesophilic organism. We observed that the high thermostability of *Pp*Cel45A (t_1/2 _of 6 h at 80°C) decreased with the removal of CBMs and glycosylated linkers (t_1/2 _of 3.8 h for *Pp*CBM1^5^:GH and t_1/2 _of 3.2 h for *Pp*GH). Controversial data are reported in the literature about factors affecting GH thermostability. The comparison of catalytic module alone with catalytic module fused to CBM showed either improvement of thermostability [36], or on the contrary appeared to have a negative impact [37]. In our study, results strongly suggest that the presence of linkers and CBMs is responsible for the protection of *Pp*Cel45A catalytic module against thermal denaturation.

Removal of CBM moieties from cellulases or hemicellulases generally reduces their hydrolytic activity on insoluble substrates, whereas their activity remains unchanged on soluble substrates [38,39]. In this study, we have shown that the presence of five CBMs in *Pp*Cel45A was not an advantage for crystalline cellulose binding and hydrolysis. Instead of diminishing catalytic module capacities, the deletion of four CBMs favoured endoglucanase activity, as shown by the measurement of (i) specific activities, (ii) end-products formation, and (iii) binding parameters. The deletion of the five CBMs diminished the catalytic properties of *P. pastoris *endoglucanase only at 65°C, which is probably the consequence of a lower thermostability of *Pp*Cel45A. Molecular modeling of individual CBMs showed that all of them share similar conformation with a flat surface containing the three key aromatic residues involved in crystalline cellulose binding (not shown). Thus, the presence of these five CBMs appended to the catalytic module of *P. pastoris *endoglucanase remains enigmatic. Our hypothesis is that the steric hindrance of five CBMs could prevent the exhibition of the flat surface required for interaction with cellulose chains, limiting enzyme action. Upon deletion of four CBMs, the enzyme probably recovers an extended conformation due to the remaining glycosylated linker, thus more adapted to efficient degradation of crystalline cellulose.

The present study has revealed the specific features of the first characterized endo β-1,4 glucanase natively issued from yeast (Saccharomycetales order). The thermostability properties of this endoglucanase could be of great interest for lignocellulosic biomass applications related to the saccharification and fermentation processes. In these industrial processes aiming at decreasing conversion cost, a high dry matter content is necessary. Integration of a high temperature pre-hydrolysis step in the process sequence leading to rapid liquefaction is an attractive way to overcome the problems associated with high initial solids, i.e. poor mixing and mass transfer, high viscosity [33]. In these processes, high temperature enzymatic liquefaction is necessary as a pre-hydrolysis step to decrease viscosity and requires thermostable (hemi)cellulases. Endoglucanases were shown to have a superior ability to rapidly reduce the viscosity of pretreated wheat straw [40]. The high thermostability of *Pp*Cel45A would be an appropriate tool for an application in such process. Consolidated bioprocessing is also an approach developed for competitive production of soluble sugars from lignocellulosic biomass in which the hydrolytic enzymes are simultaneously produced *in situ *by the fermentative microorganism [41]. The dominant strategy for engineering an efficient biocatalyst is to express cellulolytic and hemicellulolytic enzymes in *Saccharomyces cerevisae*, but expression of fungal and bacterial genes in *S. cerevisiae *has some limitations [42]. The utilization of enzymes from yeast such as *P. pastoris *endoglucanase, could also be a promising way to optimize the ethanol production process without the addition of exogenous enzymes.

## Materials and methods

### Materials and strains

*P. pastoris *X-33 and GS115 strains, *Escherichia coli *DH5α competent cells were purchased from Invitrogen (Cergy-Pontoise, France). Glucose, cellobiose, sodium carboxymethyl cellulose (CMC-Na, medium viscosity), hydroxyethyl cellulose (HEC), Avicel PH101, chitin, 4-nitrophenyl β-D-glucopyranose (pNP-glu), 4-nitrophenyl β-D-cellobioside (pNP-cel), and 4-nitrophenyl β-D-lactopyranose (pNP-lac) were purchased from Sigma (Lyon, France). Barley β-glucan, lichenan, Konjac glucomannan (KGM), mannan, and cello-oligosaccharides ranging from 3 to 6 residues (G3-G6) were purchased from Megazyme (Wicklow, Ireland). Walseth cellulose was provided by N. Lopes-Ferreira (IFPEN, Paris, France).

### Bioinformatics analysis

The catalytic domain of 55 modularly annotated fungal members of family GH45 was extracted from the CAZy database (September 2011, [8]). All sequence alignments were performed with the multiple sequence alignment program MUSCLE v3.7 [43] under default conditions. Using an in-house modified version of Jalview [44], we calculated a maximum-likelihood distance matrix using the JTT matrix [45] and submitted it to hierarchical clustering using the Ward method [46]. The figure was edited using Dendroscope 2.6.1. [47].

### Culture of *P. pastoris *GS115 on Walseth cellulose plates

*P. pastoris *GS115 was grown overnight at 30°C on agar plates containing minimal agar medium supplemented with either 2% dextrose or 6% walseth cellulose.

### Construction of expression plasmids

Genomic DNA was purified from *P. pastoris *GS115 using NucleoSpin**^® ^**Plant II kit (Macherey-Nagel, Düren, Germany). The DNA sequence [Genbank:PAS_chr4_0643] corresponding to [Genbank:CAY71902] was amplified by polymerase chain reaction (PCR) using specific primers (Table 5). *Eco*RI and *Xba*I restrictions sites were introduced respectively at the 5' and 3' end of the amplified fragments. DNA amplification was carried out with Expand High Fidelity polymerase (Roche Applied Science, Meylan, France) through 35 cycles of denaturation (20 seconds at 94°C), annealing (40 seconds at 55°C), and elongation (2 minutes at 72°C). DNA fragments were sub-cloned into pCRII-TOPO-TA vector (Invitrogen, Cergy-Pontoise, France) and transformed into *E. coli *DH5α competent cells (One shot chemically competent DH5α, Invitrogen) according to manufacturer's instructions. Recombinant plasmids were purified (Wizard plus SV miniprep kit, Promega Charbonnières, France) and sequenced. Amplified fragments were digested by *Eco*RI and *Xba*I and ligated to the *Eco*RI-*Xba*I digested pPICZαA vector (Invitrogen), in frame with both the α-factor and C-term-(His)_6-_tag encoding sequences. The resulting recombinant expression plasmids were linearized with *Pme*I and transformed into *P. pastoris *X-33 by electroporation as described in Couturier *et al*, 2011. The transformants were selected on YPDS plates (1% yeast extract, 2% peptone, 2% glucose, 1 M sorbitol, 2% agar) containing zeocin concentrations varying from 100 to 1000 μg/ml. After three days at 30°C, zeocin-resistant *P. pastoris *transformants were screened for methanol utilization (Mut^S ^phenotype) on MD (3.4% YNB, 1% ammonium sulphate, 2% glucose, 0.4 μg/ml D-biotin, 2% agar) and MM (3.4% YNB, 1% ammonium sulphate, 0.4 μg/ml D-biotin, 0.5% agar) plates. 10 Mut^S ^colonies were used for protein expression as follows: cultures were carried out in 10 ml BMGY (phosphate buffer 100 mM pH6, 1% yeast extract, 2% peptone, 3.4% YNB, 1% ammonium sulphate, 1% glycerol, 0.4 μg/ml D-biotin) in 50 ml loosely capped tubes at 30°C and shaked at 200 rpm, and expression was induced by transferring the cells into 2 ml BMMY (phosphate buffer 100 mM pH6, 1% yeast extract, 2% peptone, 3.4% YNB, 1% ammonium sulphate, 0.4 μg/ml D-biotin, 3% v/v methanol). The medium was supplemented daily with 3% methanol, and after 4 days the cultures were centrifuged and supernatants were analyzed by SDS-PAGE to select the best transformant for each enzyme.

**Table 5:** Oligonucleotide sequences of the primers used in this study.

Orientation and primer	Sequence (5'-3')
Forward	
*GH45CBMPiEcoF*	GAATTCGCTCAAGCTGAAACTGCATCCC
*GH45PiEcoF*	GAATTCGGAGACTTTGAGACAATCCCC
*GH45CBM1EcoF*	GAATTC GATGTCCCACTTTGGGGCCAA

Reverse	
*CBMPiXbaR*	TCTAGACCTGATGAAGTCGTTTCCTC
*GH45CBMPiXbaR*	TCTAGACCTTCGTCCGTACGAGCACA

The restriction sites added to insert genes into pPICZαA are underlined.

### Recombinant enzyme production and purification

For each enzyme, large-scale production (2 litres) was performed in 500 ml baffled flasks, each containing 100 ml of BMGY medium. Cells were grown overnight at 30°C and 200 rpm, and recovered by centrifugation the following day. Pellets were pooled and resuspended in 400 ml (100 ml of BMMY in 500 ml flasks), and induction was carried out for 4 days. Supernatant was then collected and processed as described in [27]. It was filtered through 0.2 μm membranes (Durapore GV membrane filters, 0.22 μm, Millipore, Molsheim, France), and concentrated (Vivaspin 10 kDa, PES, Sartorius, Palaiseau, France). A nickel chelate (GE Healthcare, Buc, France) column (His trap HP column, GE Healthcare, 0.7 cm × 5 cm) was connected to a FPLC Äkta (GE Healthcare) and equilibrated with the equilibration buffer (Tris-HCl 50 mM pH 7.8, NaCl 150 mM, imidazole 10 mM) before purification. The concentrated supernatant was diluted in the equilibration buffer and loaded onto the column at 4°C. The enzyme was eluted with Tris-HCl 50 mM pH 7.8, NaCl 150 mM, imidazole 150 mM, and the eluate was concentrated and dialysed with 50 mM sodium acetate buffer pH5 as described in [27].

### Biochemical characterization

SDS-PAGE was performed in 10% (w/v) polyacrylamide gels (Bio-Rad, Marne-la-coquette, France) using a Pharmacia LMW electrophoresis calibration kit (GE Healthcare). Proteins were detected by staining the gel with Coomassie blue. Protein concentrations were determined by the Folin-Lowry method [48] using Folin Ciocalteu's phenol reagent (Sigma-Aldrich) and bovine serum albumin as a standard.

### Enzymatic activity assays

Unless otherwise indicated, assay mixtures were prepared in citrate phosphate buffer pH 4.8. The activities of enzymes towards different cellulose derivatives (CMC, HEC, Avicel, Walseth cellulose) and complex polysaccharides (barley β-glucan, lichenan, chitin, Konjac glucomannan and mannan) were determined. All substrates excepting chitin were purchased as powders. To determine the activity towards chitin, dry chips of substrate were ground using a cutting mill (A11 cutting mill, IKA-Werke, Staufe, Germany) and further comminuted using a ball-mill grinder (MM 400, Retsch, Haan, Germany). A 0.2 mm selection screen was used to collect the finest fraction. To determine specific activity towards the various substrates, the DNS method was used as described in [49]. Briefly, 100 μl of suitably diluted enzyme (2 μg of *Pp*CBM1^1-5 ^or equimolar amounts of *Pp*GH, *Pp*CBM1^5^:GH, and *Pp*Cel45A) were mixed with 100 μl of 2% substrate in 50 mM citrate phosphate buffer pH 4.8, or 100 μl buffer containing one disk of filter paper Whatman n°1. The reactions mixtures were incubated at 45°C for various time lengths. The reaction was terminated by the addition of 300 μl 1% w/v dinitrosalicylic reagent, and samples were heated at 95°C for 10 minutes. The reaction mixtures were cooled at room temperature, and 80 μl were transferred to a microtiter plate. Reducing sugar release was determined by measuring the absorbance at 540 nm, and a glucose standard curve (0 to 10 mM) was used to calculate the release of sugars. One unit of enzyme was defined as the amount of protein releasing 1 μmol of sugar per minute. To take the different size of the four constructions into account, we chose to express our specific activities as U per μmol enzyme or U per nmol enzyme. Activity towards *p*NPCel, *p*NPLac, and *p*NPGlu was determined by measuring the release of *p*-nitrophenol in 50 mM citrate phosphate buffer, pH 4.8 after 2 hours incubation at 45°C. Either 2 μg of *Pp*CBM1^1-5 ^or equimolar amounts of *Pp*GH, *Pp*CBM1^5^:GH, and *Pp*Cel45A were used in a final reaction volume of 110 μl. Following the incubation, the same volume of 1 M sodium carbonate, pH 9 was added to terminate the reaction, and the release of *p*-nitrophenol was quantified at 410 nm using the molar extinction coefficient for *p*NP of 18300 M^-1^.cm^-1^. One unit of enzyme activity was defined as the amount of protein that released 1 μmol *p*-nitrophenol per minute.

### Effect of pH and temperature

The apparent optimal temperature was estimated in the range 4-85°C (10 temperature tested) using CMC at 1% in 50 mM citrate phosphate buffer, pH 4.8 hydrolyzed for 30 minutes at 45°C. The apparent optimal pH was estimated in the range 3-10 (9 pH tested: pH 3-6 citrate phosphate buffer, pH 7-8 phosphate buffer, pH 9-10 carbonate-bicarbonate buffer) under the conditions used. For determination of thermal stability, the purified proteins were incubated at 65 or 80°C for various time lengths (30 minutes to 48 hours), and the residual activity towards CMC was subsequently determined as described above.

### Determination of dissociation constant and carbohydrate binding capacity

For the binding assay, the reaction mixture contained 5 mg of Avicel PH101 and 40 to 200 μg of protein in a final volume of 200 μl of citrate phosphate buffer (pH 4.8). All assays were carried out in triplicates. For each assay, the mixture was incubated at 4°C for 1 h with vertical rotation (24 rpm). Substrate was removed by filtration and the concentration of unbound protein ([UB], μM) in the supernatant was measured by the Folin-Lowry method. The bound protein concentration ([B], μmol per g of substrate) was determined by substracting [UB] from the total protein concentration. Adsorption parameters were determined based on typical double-reciprocal plots using the equation [B] = [UB] × [B]_max_/(*K_d _*+ [UB]), where *K_d _*(μM) and [B]_max _(μmol per g of substrate) are the equilibrium dissociation constant and the maximum amount of protein bound, respectively [50].

### Analysis of sugar release by HPAEC-PAD

Monosaccharides and oligosaccharides generated after hydrolysis of cello-oligosaccharides (glucotetraose G4, glucopentaose G5, and glucohexaose G6) and cellulose derivatives (CMC and Avicel) were analyzed by high performance anion exchange chromatography (HPAEC) coupled with pulsed amperometric detection (PAD) (ICS 3000; Dionex, Sunnyvale, USA) equipped with a carbo-Pac PA-1 analytical column (250 × 4 mm). 20 μl of suitably-diluted enzyme were incubated at 40°C for various time lengths with 180 μl of 1 mM substrate in 50 mM acetate buffer pH 4.8. 10 μl of enzymatic reactions were stopped by the addition of 90 μl of 100 mM NaOH before injection (5 μl) into the HPAEC system. Elution was carried out in 130 mM NaOH using a 25-min linear gradient program from 100% A (130 mM NaOH) to 60% A and 40% B (NaOAc, 500 mM; NaOH, 130 mM). Calibration curves were plotted using β-1,4-cello-oligosaccharides as standards from which response factors were calculated (Chromeleon program, Dionex) and used to estimate the amount of products released. The specificity constants were calculated using the Matsui equation [51,52].

## Abbreviation list

GH: glycoside hydrolase; CBM: carbohydrate-binding module; CMC: carboxymethyl cellulose; DNS: dinitrosalycilic acid; HEC: Hydroxyethyl cellulose; pNP: paranitrophenol; *Pp*Cel45A: full-length *P. pastoris *GH45 endoglucanase; *Pp*CBM1^5^:GH: catalytic module with its proximal CBM1; *Pp*GH: catalytic module; *Pp*CBM1^1-5^: five CBM1 modules without catalytic module.

## Competing interests

The authors declare that they have no competing interests.

## Authors' contributions

MC participated in the design of the study, carried out most of the bench work and drafted the manuscript. JF and MH helped to carry out experimental work. PMC carried out the phylogenetic analysis. LLM, DN and PMC participated in the coordination of the study and proofread the manuscript. JGB conceived of the study, participated in its design and coordination and helped to draft the manuscript and submitted the manuscript. All authors read and approved the final manuscript.
